# Sex Differences in Kidney Transplantation: Austria and the United States, 1978–2018

**DOI:** 10.3389/fmed.2021.800933

**Published:** 2022-01-24

**Authors:** Sebastian Hödlmoser, Teresa Gehrig, Marlies Antlanger, Amelie Kurnikowski, Michał Lewandowski, Simon Krenn, Jarcy Zee, Roberto Pecoits-Filho, Reinhard Kramar, Juan Jesus Carrero, Kitty J. Jager, Allison Tong, Friedrich K. Port, Martin Posch, Wolfgang C. Winkelmayer, Eva Schernhammer, Manfred Hecking, Robin Ristl

**Affiliations:** ^1^Clinical Division of Nephrology & Dialysis, Department of Internal Medicine III, Medical University of Vienna, Vienna, Austria; ^2^Department of Epidemiology, Center for Public Health, Medical University of Vienna, Vienna, Austria; ^3^Center for Medical Statistics, Informatics and Intelligent Systems, Medical University of Vienna, Vienna, Austria; ^4^Department of Internal Medicine 2, Kepler University Hospital, Johannes Kepler University Linz, Linz, Austria; ^5^Arbor Research Collaborative for Health, Ann Arbor, MI, United States; ^6^School of Medicine, Pontificia Universidade Catolica do Parana, Curitiba, Brazil; ^7^Austrian Dialysis and Transplant Registry, Rohr, Austria; ^8^Department of Medical Epidemiology and Biostatistics, Karolinska Institutet, Stockholm, Sweden; ^9^European Renal Association - European Dialysis and Transplant Association Registry, Department of Medical Informatics, Academic University Medical Center, University of Amsterdam, Amsterdam Public Health Research Institute, Amsterdam, Netherlands; ^10^Sydney School of Public Health, The University of Sydney, Sydney, NSW, Australia; ^11^Section of Nephrology, Baylor College of Medicine, Selzman Institute for Kidney Health, Houston, TX, United States

**Keywords:** chronic kidney disease, dialysis, kidney transplantation, sex, gender, USRDS, ADTR

## Abstract

**Background:**

Systematic analyses about sex differences in wait-listing and kidney transplantation after dialysis initiation are scarce. We aimed at identifying sex-specific disparities along the path of kidney disease treatment, comparing two countries with distinctive health care systems, the US and Austria, over time.

**Methods:**

We analyzed subjects who initiated dialysis from 1979–2018, in observational cohort studies from the US and Austria. We used Cox regression to model male-to-female cause-specific hazard ratios (csHRs, 95% confidence intervals) for transitions along the consecutive states dialysis initiation, wait-listing, kidney transplantation and death, adjusted for age and stratified by country and decade of dialysis initiation.

**Results:**

Among 3,053,206 US and 36,608 Austrian patients starting dialysis, men had higher chances to enter the wait-list, which however decreased over time [male-to-female csHRs for wait-listing, 1978–1987: US 1.94 (1.71, 2.20), AUT 1.61 (1.20, 2.17); 2008–2018: US 1.35 (1.32, 1.38), AUT 1.11 (0.94, 1.32)]. Once wait-listed, the advantage of the men became smaller, but persisted in the US [male-to-female csHR for transplantation after wait-listing, 2008–2018: 1.08 (1.05, 1.11)]. The greatest disparity between men and women occurred in older age groups in both countries [male-to-female csHR for wait-listing after dialysis, adjusted to 75% age quantile, 2008–2018: US 1.83 (1.74, 1.92), AUT 1.48 (1.02, 2.13)]. Male-to-female csHRs for death were close to one, but higher after transplantation than after dialysis.

**Conclusions:**

We found evidence for sex disparities in both countries. Historically, men in the US and Austria had 90%, respectively, 60% higher chances of being wait-listed for kidney transplantation, although these gaps decreased over time. Efforts should be continued to render kidney transplantation equally accessible for both sexes, especially for older women.

## Introduction

According to the United States Renal Data System (USRDS) Annual Data Reports from the years 1994 (1) to 2018 (2), and at least six non-USRDS based, original articles from the United States (3–8), women with kidney failure requiring kidney replacement therapy (KRT), formerly entitled end stage kidney disease (ESKD) (9), have lower kidney transplant rates than men every year. This observation has been placed in context with gender disparity (10, 11). Compared with US men, US women are also more frequently living kidney donors (12–14). Systematic analyses from outside the United States, however, are scarce (15, 16), hindering international comparisons.

The absolute numbers of deceased and living donor kidney transplantations between the sexes should not be directly compared, as they have to be interpreted relative to the underlying dialysis population. Describing the relative sex proportions is indispensable because the dialysis population is dominated by men, at an approximate, historically consistent rate (17–20) of 60 to 40 percent (21, 22). Realizing that kidney transplantation is a stepwise process is another important prerequisite for adequately interpreting sex differences in transplantation, because wait-listing may be influenced by gender disparities (23), while sex differences in transplantation rates after wait-listing have previously been explained by biological factors, specifically higher levels of preformed antibodies among women (24). Hence, besides transplant rates alone, wait-listing rates represent an important factor in measuring fair organ distribution in kidney transplantation.

Austria is a central European country with a population of 9 million (25), with a socially funded health insurance model, in contrary to the federal and out of pocket health insurance system of the US. Austria participates in the Eurotransplant donor organ allocation system (26) and has an efficient kidney transplant (and dialysis) registry with consistent follow-up (19, 27). In the US all dialysis patients and kidney transplantations are documented by the US Renal Data System (USRDS) (28). In the present analysis, we aimed at filling part of the international knowledge gap on sex differences in kidney transplantation by investigating wait-listing and kidney transplantation rates in the US and Austria, between 1978 and 2018. Our aim was to determine the evidence, if any, for sex disparities, past and present, and to compare trends in two countries with different health care models (2).

## Materials and Methods

### Origin of the Study Population and Data Sources

In the US, all patients who start dialysis or receive kidney transplantation, regardless of insurance coverage and age, are documented in the US Renal Data System (USRDS), which is maintained since 1960 and made available to the nephrological community (28). The Austrian Dialysis and Transplant Registry (ADTR) is based on the voluntary cooperation of all 79 Austrian medical centers which cover the Austrian territory and offer kidney replacement therapy by hemodialysis or peritoneal dialysis and/or pre-KRT care and/or post-transplant care. In practice, these centers most often operate functional dialysis units, the majority of which (N = 51) are hospital-based (29). The Austrian medical centers also register their patients on the wait-list for kidney transplantation in one of the four transplant centers. All patients receiving hemodialysis or peritoneal dialysis and all kidney transplant recipients in Austria from the year 1964 forward have been entered into the ADTR database. For the present analysis, data from the ADTR were merged with the Eurotransplant database, a non-profit organization which was established in 1969 and is responsible for encouraging and coordinating donor organ allocation across 8 European countries, including Austria (30).

In the ADTR, pre-emptive transplantation can be deduced when a patient appears as having been transplanted without having a prior record as a dialysis patient (these patients were excluded from the present analysis, as further specified below). Similarly, in the USRDS data both the starting date of dialysis as well as the date of the first kidney transplantation are available, hence pre-emptive transplantation can be excluded in the same manner. In both countries, no age-restrictions regarding eligibility for kidney transplants are in place. Dates of dialysis initiation, wait-listing, transplantation and death were available in the same manner for both countries.

In the Austrian data, the precise date of wait-listing was documented only for those patients who subsequently received a donor organ. However, for all wait-listed patients a consecutive registration number from the Eurotransplant system was available. Wait-listing dates for listed patients who did not get a transplant yet were estimated by interpolation based on the consecutively awarded registration number and the known wait-listing dates of transplant patients. The accuracy of the interpolation was high, as for 7493 patients with known wait-listing dates, the deviation between actual and interpolated date was less or equal to 2 days in 75% of cases and less or equal 21 days in 95% of cases. Patients with implausibly early interpolated wait-listing dates of more than 1 year before start of dialysis were excluded, as specified below, in the section on the definitions of the study population.

Data in the ADTR (29) are nearly complete (<1% of patients lost to follow-up) and were extracted from local medical records by the responsible physicians in the various Austrian medical centers, as previously described (19, 27, 31). The present study was approved by the Ethics Committee of the Medical University of Vienna (EK No. 1363/2016).

### Definitions of Study Population, Time Periods, and Key Events

**Figure 1:**
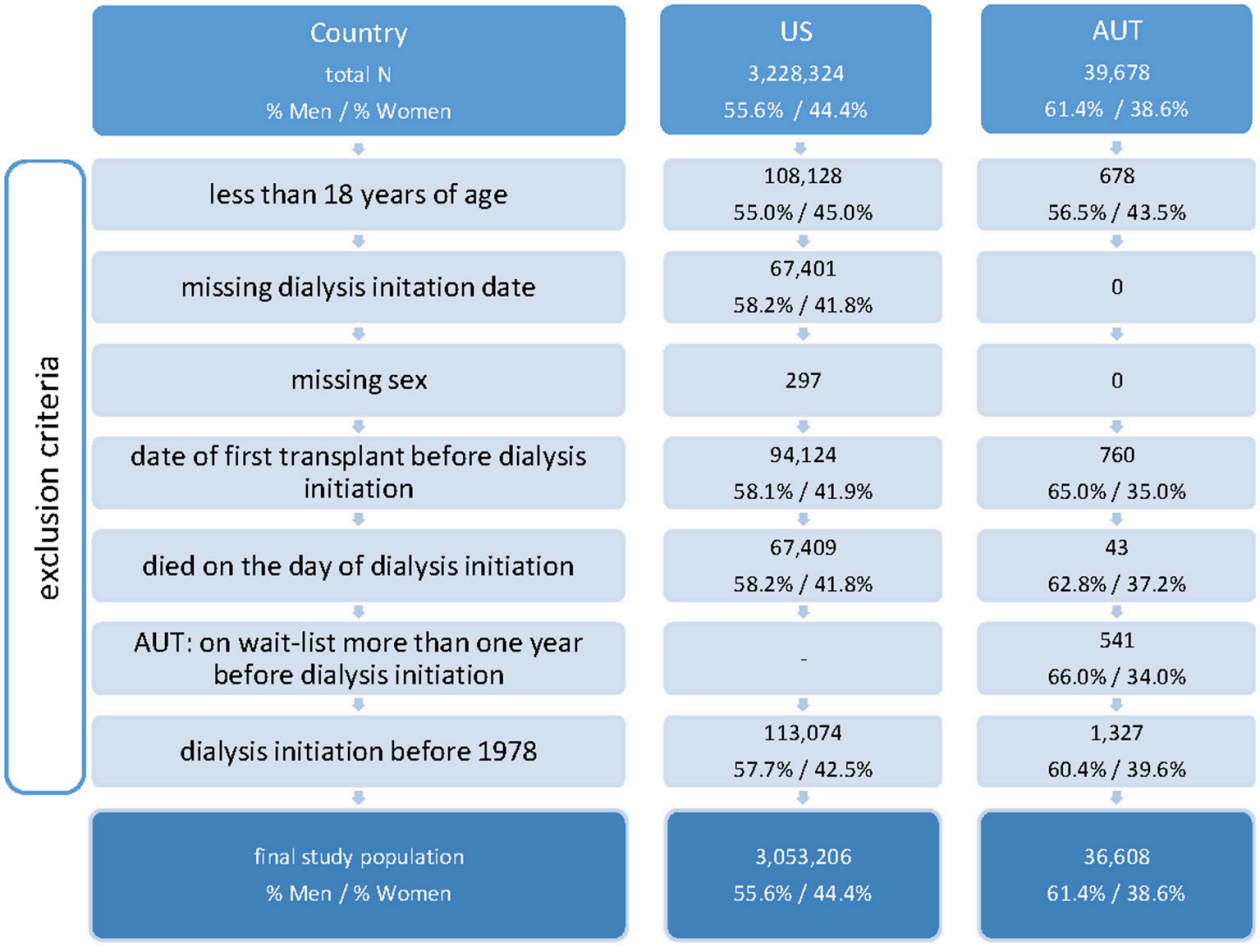
Flowchart of study population. Total number of records, number or records meeting exclusion criteria (non-exclusive), and final study population, per country.

After merging data from the ADTR and Eurotransplant, we obtained a database with records on all 39,678 patients who received kidney replacement therapy in Austria, from January 1964 through 31 August 2018. USRDS dialysis and transplant data consisted of 3,228,324 records, from May 1960 to August 2018. Due to sparse data in the early years of both datasets, for the present analysis we examined the last four decades with respect to dialysis initiation, hence USRDS and ADTR records with dialysis initiation before 1978 were excluded. Further, in both datasets we excluded patients who were aged below 18 years at dialysis initiation (US 3.3%, AUT 1.7%), those with missing data on the starting date of dialysis (US 2.1%, AUT 0%), missing information on sex (US 0.01%, AUT 0%), subjects who received a kidney transplant before dialysis initiation (US 2.9%, AUT 1.9%) and those who died on the day of dialysis initiation (US 2.1%, AUT 0.1%). Furthermore, for the Austrian data we excluded patients for whom the wait-listing date based on the Eurotransplant registration number was more than one year before the start of dialysis (1.4%). After these exclusions, the study population consisted of *N* = 3,053,026 subjects in the US (55.6% men, 44.4% women) and *N* = 36,608 in Austria (61.4% men, 38.6% women). A flowchart of the study population and the excluded data is shown in Figure 1. As sex was our exposure of primary interest, we depicted the sex distributions before and after the exclusion criteria.

To investigate time trends of sex-specific differences, we defined four periods of approximately one decade (1978–1987, 1988–1997, 1998–2007, and 2008–2018) with respect to the year of initiating dialysis. The last period encompassed 10 years and 8 months due to the last follow up date in August 2018. For analyses stratified by age at dialysis initiation, we defined three age categories, from 18 to 55 years, from 56 to 70 years, and above 70 years. The cut-points at 55 and 70 years corresponded approximately to tertiles of the patients' age distribution pooled over both countries and all decades.

We analyzed the time course of KRT, based on the recorded dates of the following events: start of dialysis, first wait-listing for transplantation, first receipt of a kidney transplant, and death.

### Statistical Analysis

For descriptive purposes, we calculated the median and interquartile range of the patients' age distribution at dialysis initiation, first wait-listing and first kidney transplantation, and absolute and relative frequencies for categorical variables, overall, and by sex. We summarized person years, mean follow-up and crude event rates per 1,000 person years of the sequential states in CKD treatment, by country and decade of dialysis initiation and per sex.

**Figure 2:**
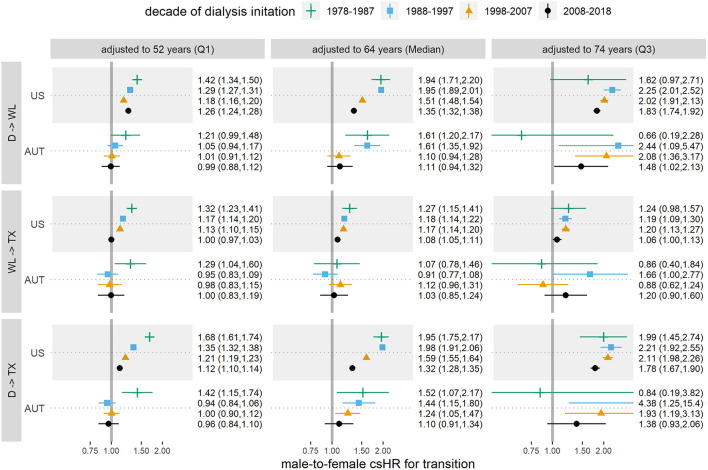
Male-to-female cause specific hazard ratios (csHRs) with 95% confidence intervals for each state transition and decade, adjusted to median age (64 years) and 25% (52 years) and 75% (74 years) age quantiles, in Austria (AUT) and the USA. (D) dialysis initiation, (WL) first entry in wait-list, (TX) first kidney transplantation. The considered transitions are dialysis to wait-list (1st row), wait-list to TX (2nd row), dialysis to TX with wait-list as intermediate state (3rd row). Age is incorporated via restricted cubic splines. Results are based on data from the ADTR/Eurotransplant (AUT) (26, 29) and USRDS (US) (28).

**Figure 3:**
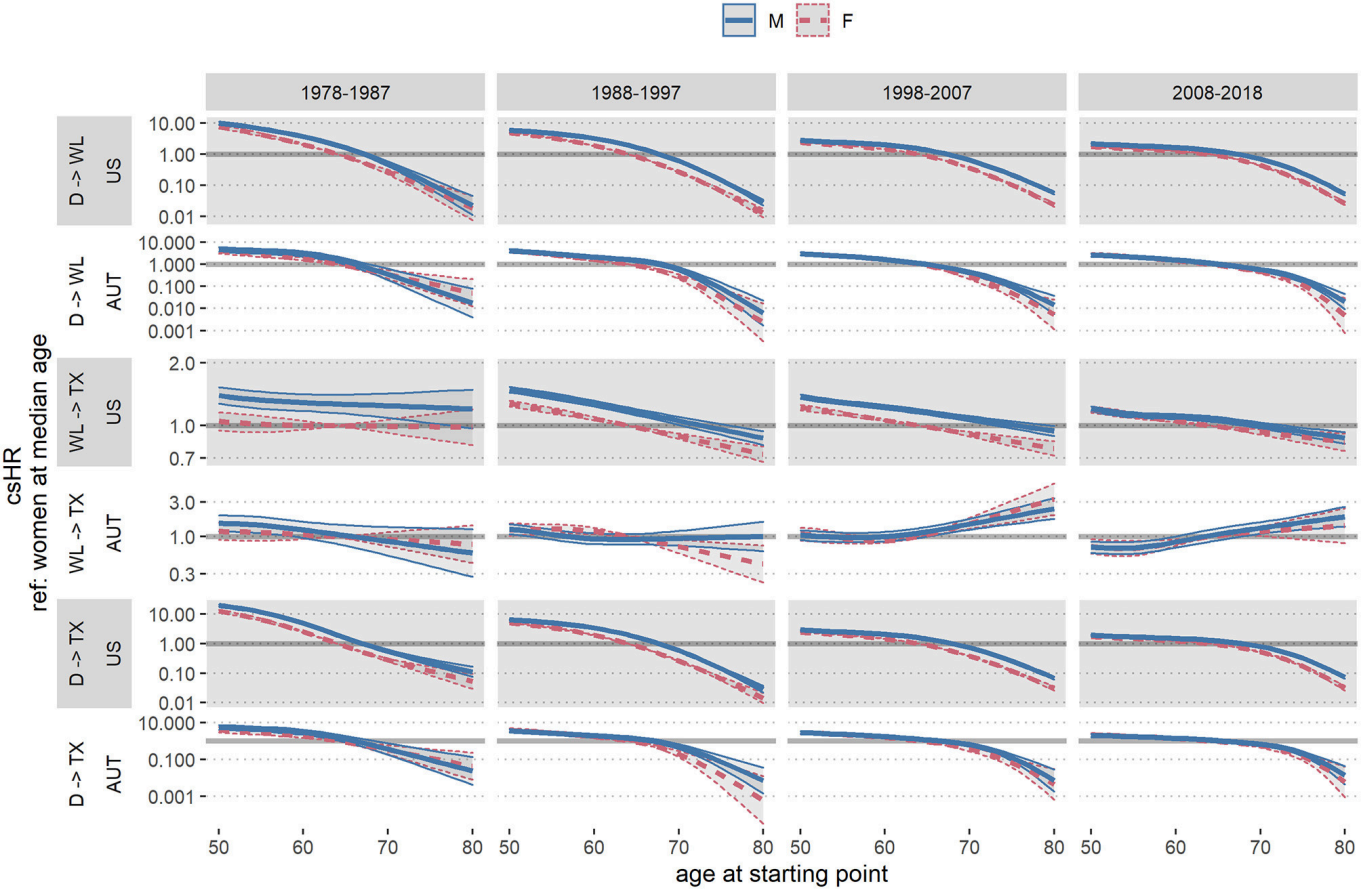
Austrian (AUT) and US cause specific hazard ratios (csHRs) and 95% confidence intervals by sex and age at the respective starting state, with median aged females as reference group, per country and decade. (D) dialysis initiation, (WL) first entry in wait-list, (TX) first kidney transplantation. Age is incorporated via restricted cubic splines. Results are based on data from the ADTR/Eurotransplant (AUT) (26, 29) and USRDS (USA) (28).

To asses sex differences in the risk (or chance) of proceeding from one state (on dialysis, wait-listed, having received kidney transplant, deceased) to another, we estimated male-to-female cause specific hazard ratios (csHRs) and the corresponding 95% confidence intervals (CIs) using Cox proportional hazard models. Cox models were fitted with the sample of all patients who had entered the respective starting state, using the individual time-point of entering the target state as baseline. The dependent variable was the time until transition to the respective target state. If applicable, the transition to another state than the considered target state of the respective model was regarded as censoring event. To allow for unbiased comparisons of the decades, all transition times were censored at 10 years. For an individual patient who started dialysis during any one decade and was subsequently followed forward for 10 years, the starting point of the analyses in some cases reached well into the next decade. To quantify the sex differences in KRT, for each transition we estimated male-to-female csHRs adjusted for age at the starting point and the interaction of age and sex. Age was incorporated via restricted cubic spline terms to account for non-linear effects. Cox models were stratified for each country and decade of dialysis initiation. We reported male-to-female csHRs adjusted to the overall median age of 64 years, as well as the 25% and 75% age quantiles (q25: 52 years, q75: 74 years) (Figure 2; Supplementary Figure 1). Further, we depicted csHRs and 95% CIs as a function of age for men and women, with median aged women as the reference group (Figure 3; Supplementary Figure 2). Note that both visualizations represent the same models, but from a different point of view. Hazard rates by the subjects' age visualize the modification of the sex effect by age.

Additionally, for each year from 1995 to 2018 we calculated the crude wait-listing and transplant rates per 100 patient years in both countries per sex, overall and within the age groups 18–55, 56–70, and 70+ years. The number of respective events were divided by the sum of observed person years within the given calendar year and multiplied by 100.

All analyses were performed using the statistical software R, version 4.0.4.

### Recording of Patient Sex

Recording of patient sex in both datasets occurred in the form of a binary variable. To our best knowledge, neither dataset differentiated between sex (male vs. female) and gender (man vs. woman) or transgender (32). Throughout the current manuscript, individuals of male and female sex are referred to as men and women, respectively, in order to remain consistent with previous work (20, 21). For reasons of legibility, hazard rates for men, divided by respective hazard rates for women are referred to as male-to-female HRs (rather than men-to-women HRs).

## Results

### Sex Differences in Patient Characteristics: Age and Type of Kidney Disease

During the study period, 3,053,206 US and 36,608 Austrian patients met our inclusion criteria (US: 55.5% men, 44.5% women, AUT: 61.4% men, 38.6% women) (Figure 1). The sex distribution in both the ADTR and USRDS did not change after excluding non-eligible subjects. In Table 1, we present time trends of patient characteristics at their start of dialysis, by country and sex. Through the decades, men who initiated dialysis were younger than women in both countries, with the differences in median age ranging from 2 to 5 years. Overall, age at dialysis initiation increased steadily over time: In 1978–1987, in the US the median age at dialysis initiation was 54.1 years for men and 55.8 years for women, while in 2008–2018 the median age at dialysis initiation was 63.3 years for men and 64.3 years for women. In Austria the respective median ages were 49.5 years (men) and 51.6 years (women) in 1978–1987, and 64.9 years (men) and 66.3 years (women) in 2008–2018. The distribution of the type of kidney disease that necessitated KRT also changed over time. In both countries, glomerulonephritis as one of the main drivers of KRT decreased and diabetes and hypertension became the most common primary diseases in the more recent decades. In both countries, the proportion of women among all patients initiating dialysis was relatively stable. In the US, relative frequencies ranged from 42.4 to 46.7%. In Austria, in the first three decades the relative frequencies ranged from 39.2 to 42.4%, while in the most recent decade the proportion of women was somewhat smaller (34.4%).

**Table 1:** Patient characteristics who initiated dialysis in the US and Austria (AUT), by decade.

**Country**		**1978–1987**		**1988–1997**		**1998–2007**		**2008–2018**	
**AUT**		**M**	**F**	**M**	**F**	**M**	**F**	**M**	**F**
		***N*** **=** **2,498 (57.6%)**	***N*** **=** **1,839 (42.4%)**	***N*** **=** **4,755 (57.6%)**	***N*** **=** **3,505 (42.4%)**	***N*** **=** **7,061 (60.8%)**	***N*** **=** **4,544 (39.2%)**	***N*** **=** **8,136 (65.6%)**	***N*** **=** **4,270 (34.4%)**
	**Age at dialysis initiation**								
		49.5 (14.5)	51.6 (15.1)	56.8 (14.8)	60.1 (15.3)	62.1 (14.4)	65.0 (14.5)	64.9 (14.1)	66.3 (14.7)
	**Age at wait-listing**								
		52.1 (15.7)	54.8 (16.7)	59.1 (15.8)	62.8 (16.5)	64.9 (15.1)	68.2 (15.5)	67.2 (14.5)	68.8 (15.1)
	**Age at first transplant**								
		53.1 (15.3)	55.8 (16.2)	60.1 (15.2)	63.6 (15.9)	65.7 (14.5)	68.8 (14.8)	67.6 (14.1)	69.1 (14.7)
	**Primary disease (ERA group)**								
	Diabetes	337 (13.5%)	227 (12.3%)	1156 (24.3%)	927 (26.4%)	2274 (32.2%)	1494 (32.9%)	2352 (28.9%)	1106 (25.9%)
	Glomerulonephritis/sclerosis	941 (37.7%)	421 (22.9%)	1053 (22.1%)	448 (12.8%)	1091 (15.5%)	457 (10.1%)	971 (11.9%)	373 (8.7%)
	Hypertension	79 (3.2%)	30 (1.6%)	280 (5.9%)	105 (3%)	625 (8.9%)	297 (6.5%)	865 (10.6%)	400 (9.4%)
	Miscellaneous	347 (13.9%)	348 (18.9%)	655 (13.8%)	755 (21.5%)	957 (13.6%)	815 (17.9%)	1362 (16.7%)	841 (19.7%)
	Polycystic kidney, adult type	157 (6.3%)	152 (8.3%)	268 (5.6%)	242 (6.9%)	361 (5.1%)	310 (6.8%)	390 (4.8%)	321 (7.5%)
	Pyelonephritis	295 (11.8%)	416 (22.6%)	343 (7.2%)	418 (11.9%)	267 (3.8%)	277 (6.1%)	262 (3.2%)	187 (4.4%)
	Renal vascular disease	72 (2.9%)	31 (1.7%)	321 (6.8%)	155 (4.4%)	760 (10.8%)	386 (8.5%)	1051 (12.9%)	519 (12.2%)
	Unknown	270 (10.8%)	214 (11.6%)	679 (14.3%)	455 (13%)	726 (10.3%)	508 (11.2%)	883 (10.9%)	523 (12.2%)
**US**		**M** ***N*** **=** **136,162 (55.1%)**	**F** ***N*** **=** **110,737 (44.9%)**	**M** ***N*** **=** **328,612 (53.3%)**	**F** ***N*** **=** **287,601 (46.7%)**	**M** ***N*** **=** **536,918 (54.6%)**	**F** ***N*** **=** **446,855 (45.4%)**	**M** ***N*** **=** **694,565 (57.6%)**	**F** ***N*** **=** **511,576 (42.4%)**
	**Age at dialysis initiation**								
		54.1 (17.1)	55.8 (16.5)	59.1 (16.5)	60.9 (15.8)	62.5 (15.6)	63.8 (15.3)	63.3 (14.8)	64.3 (14.9)
	**Age at wait-listing**								
		58.1 (16.6)	60.1 (16.2)	62.2 (16.5)	64.1 (15.9)	65.7 (15.4)	67.1 (15.3)	65.6 (14.7)	66.7 (14.9)
	**Age at first transplant**								
		57.4 (17.3)	59.7 (16.8)	62.6 (16.2)	64.5 (15.6)	66.3 (14.9)	67.7 (14.8)	66.1 (14.4)	67.1 (14.6)
	**Primary disease**								
	Cystic kidney	4,423 (3.2%)	4,104 (3.7%)	8,760 (2.7%)	7,471 (2.6%)	10,896 (2%)	9,329 (2.1%)	12,553 (1.8%)	10,366 (2%)
	Diabetes	24,695 (18.1%)	26,268 (23.7%)	102,670 (31.2%)	113,769 (39.6%)	222,766 (41.5%)	209,973 (47%)	305,433 (44%)	236,206 (46.2%)
	Glomerulonephritis	22,328 (16.4%)	14,963 (13.5%)	44,696 (13.6%)	34,524 (12%)	53,301 (9.9%)	43,096 (9.6%)	47,282 (6.8%)	38,388 (7.5%)
	Hypertension	28,961 (21.3%)	20,369 (18.4%)	92,198 (28.1%)	680,15 (23.6%)	150,151 (28%)	114,135 (25.5%)	200,308 (28.8%)	140,085 (27.4%)
	Missing cause	0 (0%)	0 (0%)	0 (0%)	0 (0%)	1 (0%)	0 (0%)	226 (0%)	114 (0%)
	Other cause	11,510 (8.5%)	10,758 (9.7%)	25,679 (7.8%)	17,741 (6.2%)	52,892 (9.9%)	36,075 (8.1%)	67,402 (9.7%)	44,816 (8.8%)
	Other urologic	2,892 (2.1%)	975 (0.9%)	6,711 (2%)	3,649 (1.3%)	10,003 (1.9%)	5,244 (1.2%)	10,279 (1.5%)	4,518 (0.9%)
	Unknown cause	7,815 (5.7%)	5,940 (5.4%)	14,488 (4.4%)	10,832 (3.8%)	22,848 (4.3%)	16,424 (3.7%)	15,796 (2.3%)	10,783 (2.1%)
	Missing	33,538 (24.6%)	27,360 (24.7%)	33,410 (10.2%)	31,600 (11%)	14,060 (2.6%)	12,579 (2.8%)	35,286 (5.1%)	26,300 (5.1%)

*Diagnosis data are displayed as absolute and relative frequencies. Ages are displayed as median (interquartile range)*.

### Sex Differences in Kidney Recipient and Donor Characteristics

Throughout the study period, 385,642 US and 9,966 Austrian patients in the study population received their first kidney transplant; in the US 61.5% and in Austria 64.9% of the transplant recipients were men. In Table 2, we summarize the respective donor characteristics for each decade. Throughout the years, the proportion of living donor kidneys increased, especially the proportion of living kidney donation from men donors. Deceased donor kidneys continued to be more frequently available from men than from women, however with decreasing tendency toward the most recent decade.

**Table 2:** Donor and recipient characteristics, by decade of dialysis initiation.

**Country**	**Donor sex**	**Donor type**	**1978–1987**		**1988–1997**		**1998–2007**		**2008–2018**	
**AUT**			**M** ***N*** **=** **1,136**	**F** ***N*** **=** **689**	**M** ***N*** **=** **1,796**	**F** ***N*** **=** **1,057**	**M** ***N*** **=** **2,020**	**F** ***N*** **=** **1,032**	**M** ***N*** **=** **1,513**	**F** ***N*** **=** **723**
	M	Cadaver	52.10%	50.10%	51.60%	51.80%	46.70%	48.60%	45.40%	44.50%
		Living	0.80%	0.60%	0.80%	1.10%	2.60%	3.30%	3.70%	6.60%
	F	Cadaver	39.70%	41.40%	39.40%	40.90%	40.40%	41.00%	35.80%	39.70%
		Living	0.80%	1.30%	1.30%	1.40%	4.90%	3.00%	12.50%	6.80%
	Unknown	Cadaver	6.50%	6.50%	5.80%	4.50%	5.10%	3.60%	2.00%	2.20%
		Living	0.10%	0.10%	1.10%	0.30%	0.30%	0.50%	0.60%	0.10%
**US**			**M** ***N*** **=** **38,581**	**F** ***N*** **=** **23,796**	**M** ***N*** **=** **66,629**	**F** ***N*** **=** **43,645**	**M** ***N*** **=** **83,881**	**F** ***N*** **=** **52,652**	**M** ***N*** **=** **57,104**	**F** ***N*** **=** **34,256**
	M	Cadaver	46.00%	45.10%	46.00%	43.80%	41.30%	39.30%	40.90%	41.20%
		Living	9.80%	9.60%	9.80%	10.80%	12.20%	13.40%	11.80%	11.50%
		Unknown	1.10%	1.00%	0.20%	0.20%	0.00%	0.00%	0.00%	0.00%
	F	Cadaver	24.60%	24.70%	29.90%	29.70%	26.90%	27.80%	25.10%	27.70%
		Living	9.40%	10.30%	12.20%	13.20%	17.70%	17.30%	20.80%	18.00%
		Unknown	0.70%	0.80%	0.20%	0.20%	0.00%	0.00%	0.00%	0.00%
	Unknown	Cadaver	4.30%	4.40%	1.00%	1.10%	1.20%	1.40%	0.90%	1.10%
		Living	1.30%	1.30%	0.30%	0.40%	0.60%	0.70%	0.50%	0.40%
		Unknown	2.70%	2.80%	0.50%	0.50%	0.10%	0.10%	0.00%	0.10%

*Results refer to the first kidney transplantation of a patient*.

### Sex Differences in the Event Course of KRT

#### Time and Age Trends in Wait-Listing, Respectively, Transplantation

Table 3 shows crude event data per country and decade for the transition from dialysis initiation to being waitlisted, from waitlist entrance to receive a transplant, and from dialysis initiation to receive a transplant (with wait-listing as intermediate step). Figure 2 reports the respective male-to-female csHRs for the transitions in CKD treatment. We observed a lower chance for women on dialysis to enter the wait-list, compared to men, albeit it increased over time. Specifically, in 1978–1987 the male-to-female csHR of getting wait-listed in the US was 1.94 [95% CI 1.71, 2.20] and decreased to 1.35 [95% CI 1.32, 1.38] in the most recent decade. In Austria, in the first decade men also had significantly higher chances of being wait-listed [1978–1987 csHR 1.61 [95% CI 1.20, 2.17)] than women, however this advantage vanished in the last two decades [2008–2018 csHR 1.11 [95% CI 0.94, 1.32)]. To visualize the effect modification by age, Figure 3 depicts the same models, but with respect to the subject's age at the starting state. The chances of wait-listing decreased with older age for both sexes. In both countries, age modified the sex-specific wait-listing chances, but the effect modification decreased throughout the decades, especially for younger patients. In the US, effect modification persisted over all ages and throughout the decades (US: p_interaction_ < 0.001 for all decades, AUT: p_interaction_ < 0.05 for all decades except for the most recent). In both countries, the advantage of men for wait-listing was more distinct in older age.

**Table 3:** Crude time-to-event data per country, decade of dialysis initiation, and sex.

			**1978–1987**	**1978–1987**	**1988–1997**	**1988–1997**	**1998–2007**	**1998–2007**	**2008–2018**	**2008–2018**
**Event**	**Country**	**Variable**	**M**	**F**	**M**	**F**	**M**	**F**	**M**	**F**
D –> WL	US	Person years	452,618	397,614	900,419	848,463	1,569,883	1,362,920	1,603,818	1,248,679
		Mean (SD) follow-up	3.33 (3.1)	3.60 (3.2)	2.80 (2.9)	3.00 (2.9)	3.00 (3.0)	3.12 (3.0)	2.42 (2.3)	2.55 (2.3)
		Events	16,847	11,434	68,527	45,739	99,507	63,903	97,914	55,229
		Events per 1,000 PY	37.2	28.8	76.1	53.9	63.4	46.9	61.1	44.2
	AUT	Person years	5,109	4,571	9,234	7,843	18,229	13,094	18,947	10,378
		Mean (SD) follow-up	2.16 (2.6)	2.60 (2.9)	2.05 (2.5)	2.34 (2.7)	2.63 (2.9)	2.93 (3.2)	2.36 (2.2)	2.47 (2.3)
		Events	1,152	704	1,945	1,076	2,186	1,099	1,913	863
		Events per 1,000 PY	225.5	154.0	210.6	137.2	119.9	83.9	101.0	83.2
WL –> TX	US	Person years	19,739	19,182	156,066	118,890	347,189	248,271	364,866	225,662
		Mean (SD) follow-up	1.88 (2.4)	2.42 (2.7)	2.11 (2.3)	2.39 (2.5)	3.06 (2.8)	3.34 (3.0)	2.83 (2.3)	2.96 (2.4)
		Events	8,209	5,876	57,490	36,928	75,717	47,011	55,497	33,241
		Events per 1,000 PY	415.9	306.3	368.4	310.6	218.1	189.4	152.1	147.3
	AUT	Person years	2,383	1,665	4,605	2,547	5,191	2,785	3,231	1,549
		Mean (SD) follow-up	1.93 (2.2)	2.18 (2.4)	2.12 (2.0)	2.09 (2.0)	2.24 (2.2)	2.40 (2.3)	1.61 (1.7)	1.69 (1.7)
		Events	1,027	616	1,711	1,032	1,949	996	1,449	695
		Events per 1,000 PY	431.0	370.0	371.5	405.2	375.5	357.6	448.5	448.6
D –> TX	US	Person years	395,685	368,027	1,019,753	940,367	1,877,517	1,580,278	1,930,922	1,446,097
		Mean (SD) follow-up	2.91 (2.9)	3.32 (3.1)	3.10 (2.9)	3.27 (2.9)	3.50 (3.1)	3.54 (3.2)	2.78 (2.4)	2.83 (2.4)
		Events	37,929	23,243	65,164	42,447	81,387	50,874	57,074	34,240
		Events per 1,000 PY	95.9	63.2	63.9	45.1	43.3	32.2	29.6	23.7
	AUT	Person years	7,365	6,148	13,759	10,339	23,306	15,827	22,134	11,905
		Mean (SD) follow-up	2.95 (2.8)	3.34 (3.1)	2.89 (2.6)	2.95 (2.7)	3.30 (2.9)	3.48 (3.1)	2.72 (2.2)	2.79 (2.3)
		Events	1,111	661	1,778	1,051	2,008	1,029	1,513	723
		Events per 1,000 PY	150.8	107.5	129.2	101.7	86.2	65.0	68.4	60.7

*Person years (PY), mean (SD) follow-up, event counts and events per 1,000 person years, by event [Dialysis (D), first wait-listing (WL) and first transplant (TX)], country and decade of dialysis initiation and per sex*.

Once on the wait-list in Austria, chances to receive a donated kidney did not differ between men and women. In the US however, men on the wait-list had significantly higher chances of receiving a kidney transplant in the past [1978–1087 male-to-female csHR 1.27 (95% CI 1.15, 1.41)], although both the age and the sex effect diminished over time [2008–2018 male-to-female csHR 1.08 (95% CI 1.05, 1.11)]. Hence, the main disparity between the sexes occurred in the initial step of entering the wait-list in both countries, and was to a smaller extent driven by unbalanced sex-specific kidney transplantation. Overall, differences between the sexes regarding wait-listing and transplantation were gradually reduced throughout the study period. However, especially for patients of older age, sex differences were still prominent in the most recent decade, as can best be noted from the male-to-female csHR adjusted to the 75% age quantile (74 years) in 2008–2018, in comparison to the adjustment for median and low age. For wait-listing in this age group, we obtained a male-to-female csHR of 1.83 [95% CI 1.74–1.92] in the US and 1.48 [95% CI 1.02–2.13] in Austria.

#### Mortality

Male-to-female csHRs for death from the two starting points dialysis initiation and wait-listing are shown in the Supplementary Table 1, Supplementary Figures 1, 2. Male-to-female mortality hazards after dialysis initiation were rather similar across sexes and decades in both countries. Overall, there were tendencies for higher mortality in men. Age-adjusted mortality after transplantation was higher for men than for women throughout most decades and consistent over age groups, although confidence bands in Austria were very wide (Supplementary Figure 1).

#### Wait-Listing and Transplant Rates per 100 Patient Years

**Figure 4:**
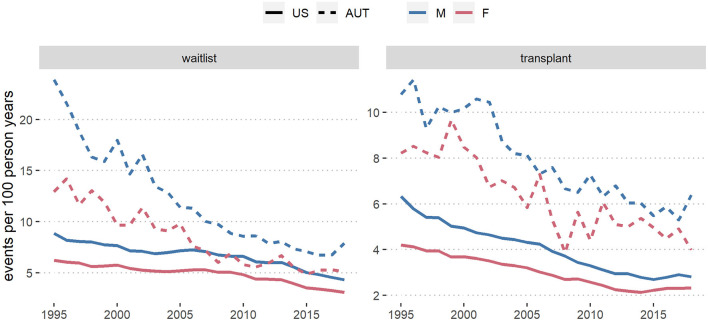
Wait-list and transplant rates per 100 dialysis patient years in the US and in Austria (AUT) from 1995 to 2018, by sex. Results are based on data from the ADTR/Eurotransplant (AUT) (26, 29) and USRDS (US) (28).

**Figure 5:**
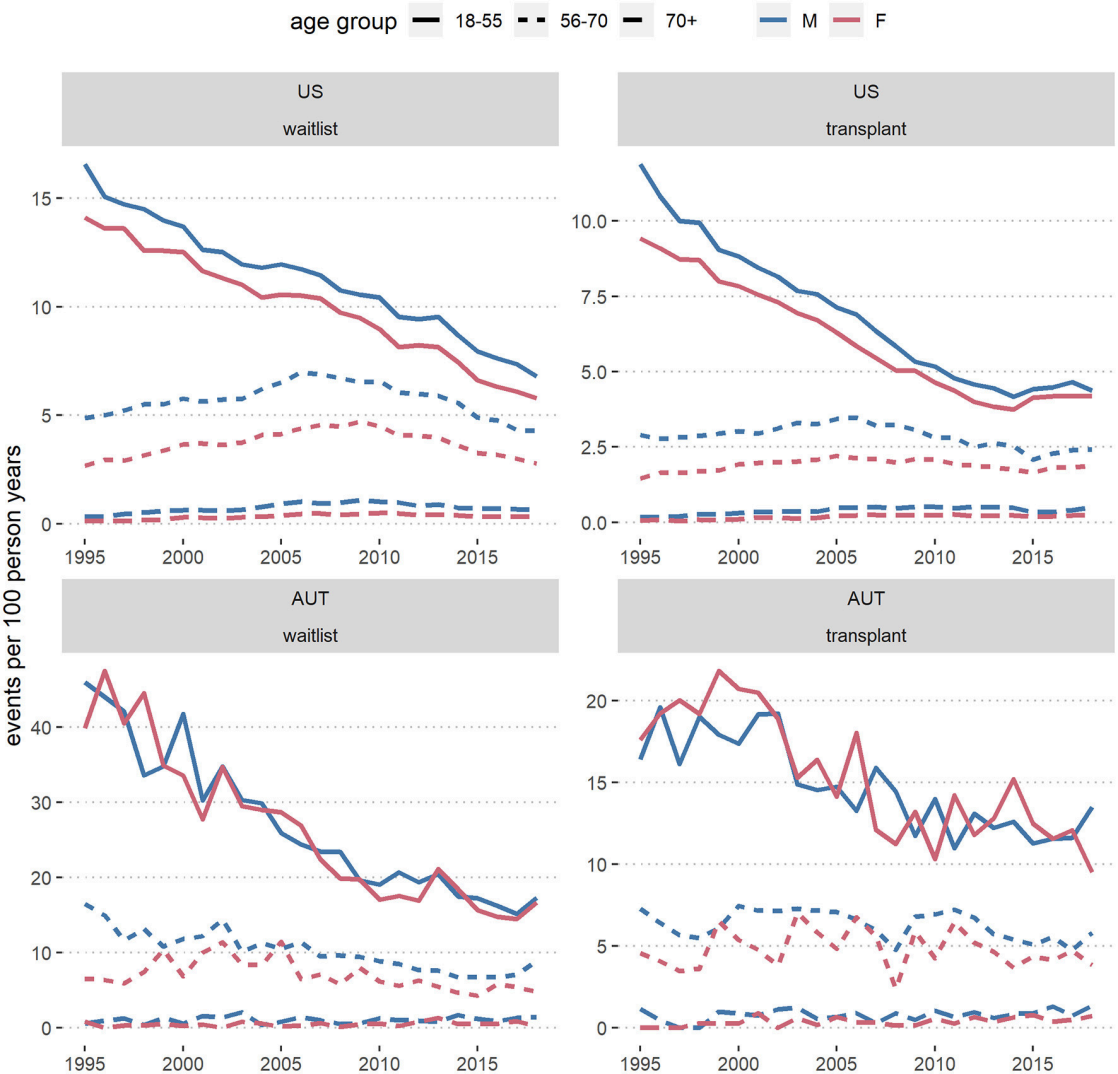
Wait-listing and transplant rates per 100 dialysis patient years in the US and Austria (AUT), from 1995 to 2018, by sex and age group. Results are based on data from the ADTR/Eurotransplant (AUT) (26, 29) and USRDS (US) (28).

In Figures 4, 5 we show crude wait-listing and transplant rates per 100 dialysis patient years, by sex and calendar year in each country, overall (Figure 4) and by age group (Figure 5). The overall crude event rates were consistently higher for men than for women and declined within the considered time frame. In the first age category (up to 55 years), wait-listing and transplant rates in Austria were similar for men and women. In all other groups, crude event rates for both wait-listing and transplantation were higher in men. This finding is in line with the age-adjusted male-to-female csHRs in Figures 2, 3. When comparing the two countries, although both the wait-listing as well as the transplant event rates were about twice as high in Austria than the US, the trends over time were very similar. However, as can be deduced from Figure 5, for the second age group (56–70 years) in the US both event rates increased from 1995 onward up to ~2010 and decreased thereafter. In Austria, event rates within this age group were rather constant.

## Discussion

In this study with historical data through 2018 from the US and Austria, we found that men had a higher chance than women of being placed on the wait-list for kidney transplantation. The age-adjusted probability for women to enter the transplant wait-list was smallest in earlier decades and among older patients, when compared to men (Figure 2). Sex differences in wait-listing decreased over time, but were still observed at all ages in the US, and especially for patients in old age. In both the US and Austria, once patients had entered the wait-list, the probability of receiving a donor organ was very similar for men and women, although in the US the advantage for men disappeared only within the most recent decade (2008–2018). Wait-listing rates by sex moved closer together in Austria than in the United States, indicating that gender or sex disparities in transplant access in Austria might have been reduced to a greater degree than in the United States.

Understanding the impact of age is important for correctly interpreting our study findings. Adjusted to the 75% age quantile, in the most recent decade the male-to-female csHR for wait-listing was 1.83 [95% CI 1.74, 1.92] in the US and 1.48 [95% CI 1.02, 2.13] in Austria (Figure 2). Further, older age was associated with a reduced probability to receive a donor organ and an increased risk of death (Figures 2, 3). In addition, women were on average older than men in the studied population (Table 1). These observations emphasize the need of accounting for age as a potential confounder in the analysis. We accomplished this task by adjusting the male-to-female csHRs within each decade for age (as continuous variable), including an interaction for sex and age. To visualize the results we chose to depict the male-to-female cause specific hazard ratio at the median age as well as 25 and 75% age quantiles (Figure 2). This summarizes the csHR at three age levels, yet the underlying model still contains age as continuous variable, represented by restricted cubic splines to account for non-linear age effects, as shown by the csHRs of men and women by age, referenced to median aged women, in Figure 3. In the most recent decade the age difference between men and women became smaller, hence the effect of age on sex differences in wait-listing may have become smaller in this decade than in earlier decades, in Austria more so compared to the US. The fact that age is an effect modifier of gender disparity in kidney transplantation has also been shown in another USRDS-based analysis (33).

To a large part, the incidence of dialysis initiation was stable throughout the study period in both countries, and consistent with previously reported sex distributions of roughly 60% men and 40% women in CKD cohorts (20). The seemingly higher proportion of men starting dialysis in Austria in the last decade (2008–2018: 65.6% men) is likely to be an artifact of the grouping over time, as previous research based on the ADTR data with a different study period and different stratification of the time intervals, did not show significant time trends by sex in dialysis initiation (19). Nevertheless, future monitoring of the ADTR should be sensible to potential trends in the sex-distribution of incident dialysis patients in Austria.

Once wait-listed, the allocation systems of the two countries theoretically do not have gender or sex-specific aspects, meaning that in principle, every listed patient has the same change to receive an organ. Yet it is known that women have higher levels of preformed antibodies, linked to pregnancy. Wolfe et al. (24) showed that sex differences in transplantation rates after wait-listing disappeared when adjusted for panel-reactive antibodies (PRA). Unfortunately, we did not have PRA data for our datasets to confirm this. In any case, as we have shown in this work, the sex differences *after* wait-listing were less pronounced than *for* wait-listing itself.

The age-adjusted male-to-female HR for death after transplantation was >1 in most decades. As the male-to-female mortality rate ratio in adults of the general population remained consistently >1 throughout age groups (21), a higher mortality risk in transplanted men compared to transplanted women might not be surprising. If men have a higher chance of being wait-listed than women, however, then the consequence might be that men who are altogether sicker than women actually receive a transplant, and the comorbidities of these patients might carry over into the post-transplant time, where men die at a higher rate than women. Consistent with this hypothesis, the age-adjusted male-to-female HR for death in the dialysis population of the present study was not as high as it was in the transplant population (although also >1 in some decades and at some ages, see Supplementary Figure 1).

Gender disparity in kidney transplantation is often mentioned in context with the perceived unfairness that women are more often donors than they are recipients of living donor transplants (15). In our analysis of US and Austrian data, and as was previously shown for the US (12–14), more living donor kidneys originated from women rather than men (Table 2). Many analyses on sex-specific differences in kidney transplantation are not based on registry data, but simply report crude (mostly living related and often single center) transplantation rates which are always shifted toward more women being donors and more men being recipients (34, 35). A wide range of explanations haven been given to the predominance of women in living kidney donation, including better health or a higher degree of responsibility in women and financial obligations of men, all of which remain speculative (11, 13, 15).

The most fundamental difference between the US and the Austria with respect to kidney disease management lies in the distinctive funding of the healthcare systems of the two countries, and thus access to dialysis and subsequent KRT. Austrian's socially funded health care system provides full coverage for its population (99.9%) (36). The majority of dialysis centers are administered by the public sector, private dialysis centers can reimburse a large part of their costs following fixed rates set by the Austrian health fund. In the US, in 2000–2016 88% of dialysis patients were treated in profit-driven facilities, 66.5% of all patients underwent dialysis at only two large, privately owned, for-profit dialysis facility chains. Gander et al. showed that patients under treatment in for-profit dialysis facilities vs. non-profit facilities had lower chances of entering the waitlist and receiving a living or deceased kidney transplant (37). In their analysis, the proportion of women in for-profit facilities was higher compared to women in non-profit facilities. It has previously been hypothesized that for-profit dialysis providers may cut costs in counseling or refrain to refer patients to KRT, since this is in contrast to their financial interests (38). A gender bias in the type of dialysis facility (for-profit vs. non-profit) thus could be a partial explanation of both, the more pronounced advantages for men in KRT in the US compared to Austria, and why gender disparities in wait-listing and transplantation still persisted in the US in the most recent decade, in contrast to Austria.

Among the limitations of this analysis, we acknowledge that it is unclear whether the sex variable was assigned by an investigator or reported by a patient. The sheer size of the dataset implies that 100% correctness cannot be assumed. Further, stratification over time did not follow any significant events in kidney disease management or policy changes, but rather split the data uniformly across the time axis, in order to reveal possible time trends. Moreover, our study cannot provide causality and therefore needs not only to be followed up in additional countries, but also by analyses of socioeconomic differences and other factors, for example obesity (39), which might explain the observed differences between the sexes, on top of age and comorbidities (33).

In summary, the present USRDS and ADTR/Eurotransplant data shed light on the sex differences for various transitions after initiation of kidney replacement therapy, with consideration of trends over four decades. Our analysis follows a recently articulated request (16) to start focusing on non-North American cohorts in examining how sex and gender affect transplantation and compared them with the US. In accordance with previous data from the US (1–8, 24, 33, 40), Canada (41), France (42), Australia (43) and Germany (44), in our analysis predominantly older women have lower access to kidney transplantation than men. Knowing the development in the US and Austria over the last four decades is informative, and this development renders it likely that gender disparity is the root cause of the observed sex differences in kidney transplantation. Future analyses should examine sex-discrepancies in dialysis providers, and also qualitatively address the perspectives of patients (45, 46) and caretakers (47), which might help establish reasons for sex and gender differences, and ways to overcome them. The items in question might include differences in generosity regarding kidney donation, differences in the perception of life, different moral values, and finally, different priorities between men vs. women, including their ability to endorse relationships, despite being affected by kidney disease.

## Data Availability Statement

The US based data reported here have been supplied by the United States Renal Data System (USRDS) and are available from USRDS upon request. The interpretation and reporting of these data are the responsibility of the authors and in no way should be seen as an official policy or interpretation of the U.S. government. The Austrian data reported here have been supplied by the Austrian Dialysis and Transplant Registry (ADTR) and are available from the Austrian Society of Nephrology upon request.

## Ethics Statement

This study was reviewed and approved by the Ethics Committee of the Medical University of Vienna (EK No. 1363/2016). Written informed consent for participation was not required for this study in accordance with the national legislation and the institutional requirements.

## Author Contributions

SH conceptualized the analysis, analyzed the data, wrote, revised, and reviewed the manuscript. TG analyzed the data. MA, JC, KJ, and AT conceptualized the analysis and reviewed the manuscript. AK, ML, SK RP-F, WW, and ES reviewed the manuscript. RK provided the data, interpreted the data, and reviewed the manuscript. FP conceptualized the analysis, discussed the data, and reviewed the manuscript. MP conceptualized the analysis. MH provided funding, conceptualized analysis, wrote, revised, and reviewed the manuscript. RR conceptualized the analysis, analyzed the data, wrote, and reviewed the manuscript. All authors contributed to the article and approved the submitted version.

## Funding

We acknowledge support from the Austrian Science Fund (grant No. KL754-B).

## Conflict of Interest

The authors declare that the research was conducted in the absence of any commercial or financial relationships that could be construed as a potential conflict of interest.

## Publisher's Note

All claims expressed in this article are solely those of the authors and do not necessarily represent those of their affiliated organizations, or those of the publisher, the editors and the reviewers. Any product that may be evaluated in this article, or claim that may be made by its manufacturer, is not guaranteed or endorsed by the publisher.
