# Supplementary material for: Sex Differences in Kidney Transplantation: Austria and the United States, 1978–2018
Source: Front Med (Lausanne). 2022 Jan 24;8:800933. doi: 10.3389/fmed.2021.800933 (PMC8819173; doi:10.3389/fmed.2021.800933)

**Supplementary Table 1. Crude time-to-event data per country, decade of dialysis initiation and sex.** Person years, mean (SD) follow-up, event counts and events per 1,000 person years, by event (dialysis (D), first transplant (TX) and death (XX)), country and decade of dialysis initiation and per sex.

|  |  |  | 1978-1987 | 1978-1987 | 1988-1997 | 1988-1997 | 1998-2007 | 1998-2007 | 2008-2018 | 2008-2018 |
| --- | --- | --- | --- | --- | --- | --- | --- | --- | --- | --- |
| Event | Country | Variable | M | F | M | F | M | F | M | F |
| D -> XX | US | Person Years | 653,939 | 526,801 | 1,455,235 | 1,223,884 | 2,383,045 | 1,901,370 | 2,140,187 | 1,573,466 |
|  |  | Mean (SD) follow-up | 4.80 (3.8) | 4.76 (3.7) | 4.43 (3.7) | 4.26 (3.5) | 4.44 (3.7) | 4.26 (3.6) | 3.08 (2.6) | 3.08 (2.6) |
|  |  | Events | 101,677 | 84,646 | 260,029 | 236,312 | 424,861 | 364,103 | 363,879 | 276,037 |
|  |  | Events per 1,000 PY | 155.5 | 160.7 | 178.7 | 193.1 | 178.3 | 191.5 | 170.0 | 175.4 |
|  | AUT | Person Years | 14,112 | 10,200 | 25,291 | 17,242 | 35,822 | 22,281 | 27,621 | 14,681 |
|  |  | Mean (SD) follow-up | 5.65 (3.9) | 5.55 (3.9) | 5.32 (3.9) | 4.92 (3.9) | 5.07 (3.9) | 4.90 (3.9) | 3.39 (2.7) | 3.44 (2.8) |
|  |  | Events | 1,570 | 1,197 | 3,215 | 2,507 | 4,934 | 3,270 | 3,791 | 2,032 |
|  |  | Events per 1,000 PY | 111.3 | 117.3 | 127.1 | 145.4 | 137.7 | 146.8 | 137.2 | 138.4 |
| TX -> XX | US | Person Years | 305,456 | 194,623 | 546,359 | 364,721 | 654,191 | 419,325 | 210,672 | 128,231 |
|  |  | Mean (SD) follow-up | 7.92 (3.2) | 8.18 (3.1) | 8.21 (3.0) | 8.36 (2.9) | 7.81 (3.0) | 7.97 (2.9) | 3.69 (2.6) | 3.74 (2.6) |
|  |  | Events | 14,831 | 8,123 | 23,711 | 14,162 | 25,903 | 14,467 | 6,043 | 3,069 |
|  |  | Events per 1,000 PY | 48.6 | 41.7 | 43.4 | 38.8 | 39.6 | 34.5 | 28.7 | 23.9 |
|  | AUT | Person Years | 8,834 | 5,592 | 14,640 | 8,937 | 16,076 | 8,381 | 5,523 | 2,798 |
|  |  | Mean (SD) follow-up | 7.78 (3.5) | 8.12 (3.2) | 8.16 (3.2) | 8.46 (3.0) | 7.96 (3.0) | 8.12 (2.9) | 3.65 (2.6) | 3.88 (2.6) |
|  |  | Events | 407 | 234 | 606 | 310 | 580 | 266 | 156 | 60 |
|  |  | Events per 1,000 PY | 46.1 | 41.8 | 41.4 | 34.7 | 36.1 | 31.7 | 28.2 | 21.4 |

**Supplementary Figure 1: Male-to-female cause specific hazard ratios (csHRs) with 95% confidence intervals for each state transition and decade, adjusted to median age (64 years) and 25% (52 years) and 75% (74 years) age quantiles, in Austria (AUT) and the USA.** (D) dialysis initiation, (TX) first kidney transplantation, (XX) death. The considered transitions are dialysis to death (1^st^ row) and TX to death with wait-list as intermediate state (2^nd^ row). Age is incorporated via restricted cubic splines. Results are based on data from the ADTR/Eurotransplant (AUT) (26, 29) and USRDS (US) (28).
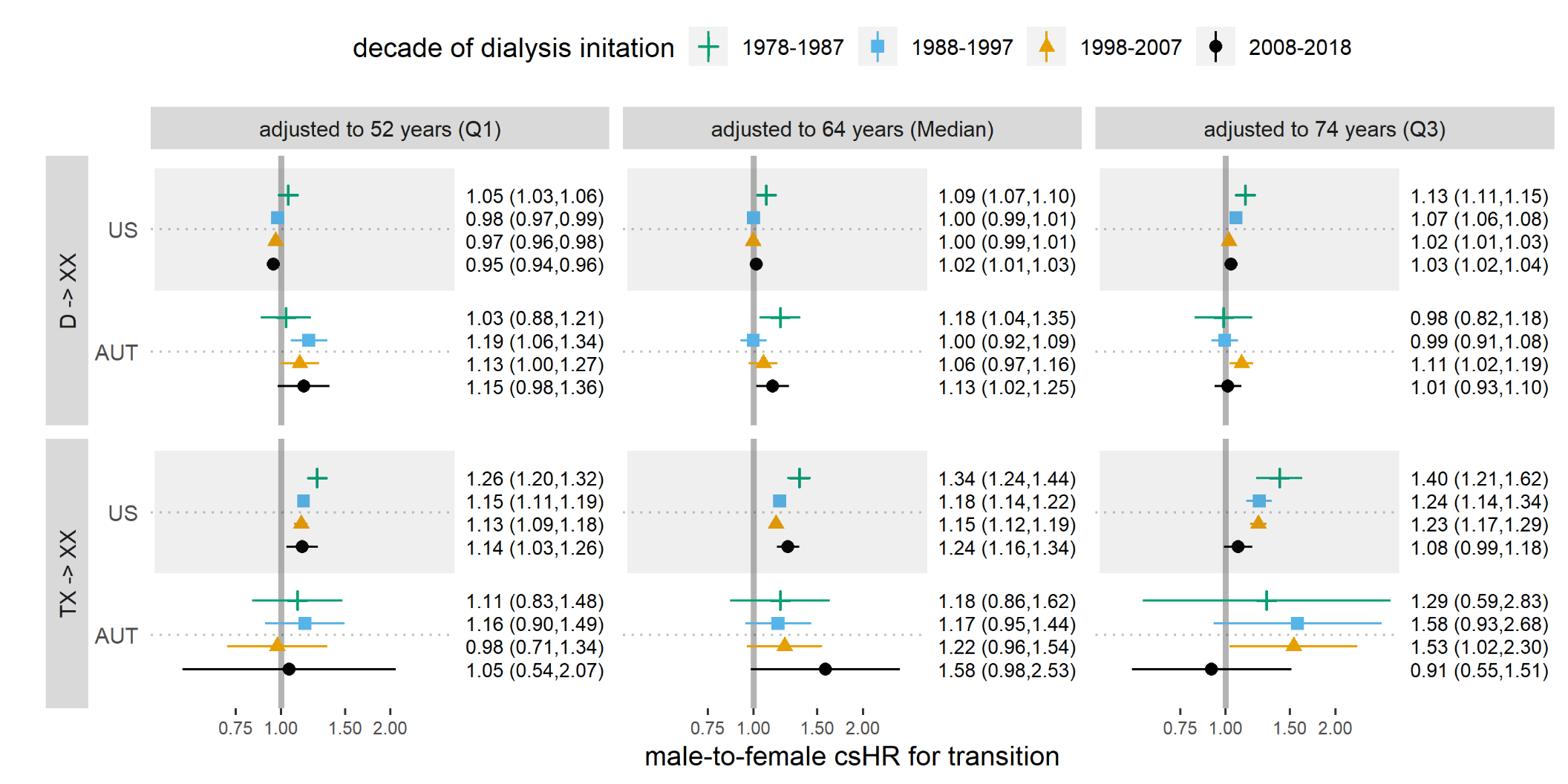


**Supplementary Figure 2: Austrian (AUT) and US cause specific hazard ratios (csHRs) and 95% confidence intervals by age and sex, with median aged females as reference group, per country and decade.** (D) dialysis initiation, (TX) first kidney transplantation, (XX) death. Age is incorporated via restricted cubic splines. Results are based on data from the ADTR/Eurotransplant (AUT) (26, 29) and USRDS (USA) (28).
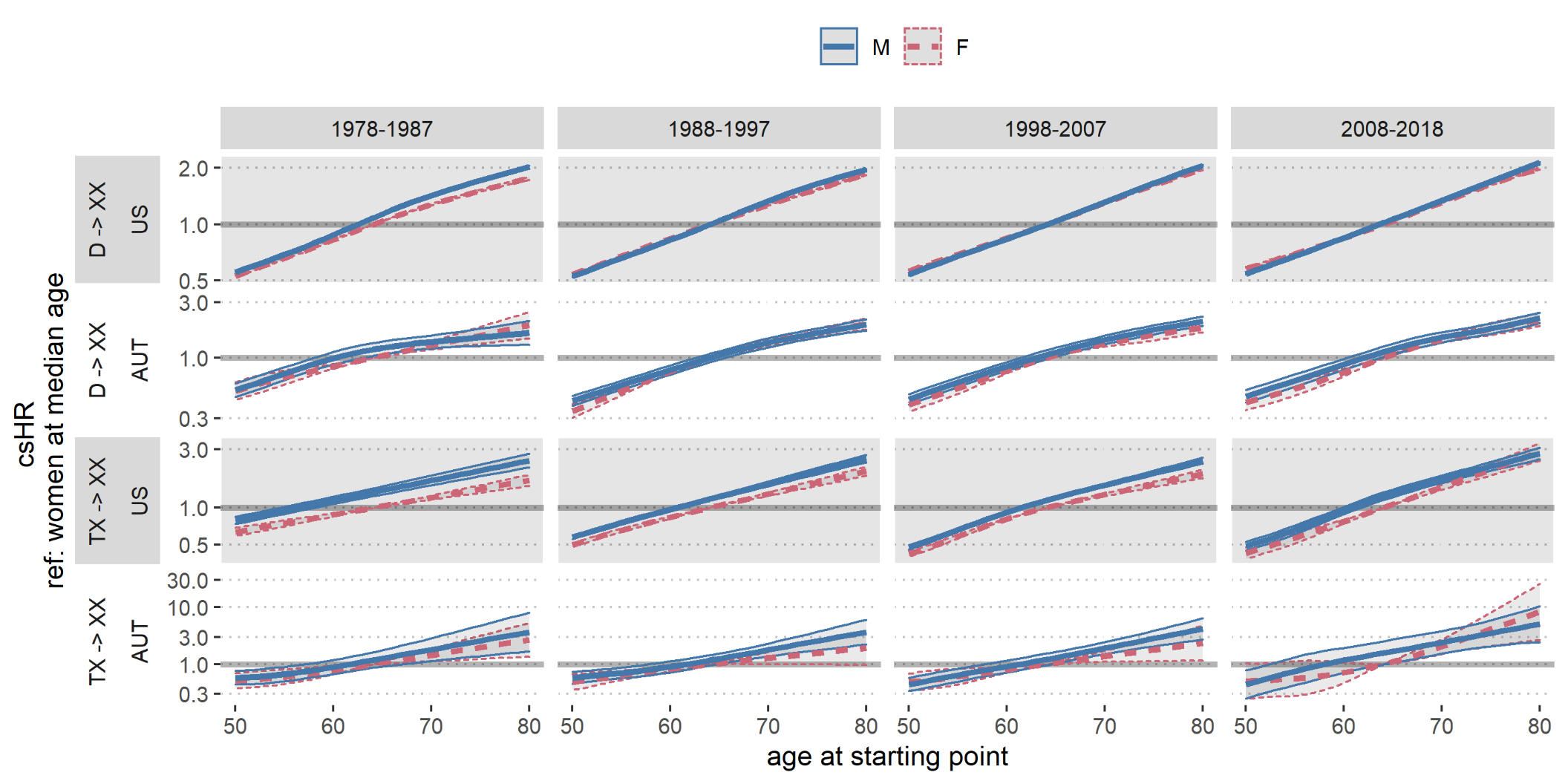

Supplement: Supplementary file 1 [file Data_Sheet_1.DOCX]
